# Rare Variants in *LRP4* Are Associated with Mesiodens, Root Maldevelopment, and Oral Exostoses in Humans

**DOI:** 10.3390/biology12020220

**Published:** 2023-01-30

**Authors:** Piranit Nik Kantaputra, Peeranat Jatooratthawichot, Ploy Adisornkanj, Panita Kitsadayurach, Massupa Kaewgahya, Bjorn Olsen, Atsushi Ohazama, Chumpol Ngamphiw, Sissades Tongsima, Timothy C. Cox, James R. Ketudat Cairns

**Affiliations:** 1 Center of Excellence in Medical Genetics Research, Faculty of Dentistry, Chiang Mai University, Chiang Mai 50200, Thailand; 2 Division of Pediatric Dentistry, Department of Orthodontics and Pediatric Dentistry, Faculty of Dentistry, Chiang Mai University, Chiang Mai 50200, Thailand; 3 Center for Biomolecular Structure, Function and Application & School of Chemistry, Institute of Science, Suranaree University of Technology, Nakhon Ratchasima 30000, Thailand; 4 Dental Department, Sawang Daen Din Crown Prince Hospital, Sakon Nakhon 47110, Thailand; 5 Dental Department, Pang Sila Thong Hospital, Kamphaeng Phet 62120, Thailand; 6 Department of Developmental Biology, Harvard School of Dental Medicine, Harvard University, Boston, MA 02115, USA; 7 Division of Oral Anatomy, Faculty of Dentistry & Graduate School of Medical and Dental Sciences, Niigata University, Niigata 951-8514, Japan; 8 National Biobank of Thailand, National Science and Technology Development Agency (NSTDA), Thailand Science Park, Pathum Thani 12120, Thailand; 9 Departments of Oral & Craniofacial Sciences, School of Dentistry, and Pediatrics, School of Medicine, University of Missouri-Kansas City, Kansas City, MO 64108, USA

**Keywords:** root malformations, torus palatinus, torus mandibularis, buccal exostoses, root anomalies

## Abstract

**Simple Summary:**

Low density lipoprotein receptor-related protein 4 (LRP4; MIM 604270) modulates WNT/β-catenin signaling, through its binding of WNT ligands, and to co-receptors LRP5/6, and WNT inhibitors DKK1, SOSTDC1, and SOST. LRP4 binds to SOSTDC1 and WNT proteins establishing a negative feedback loop between Wnt/β-catenin, Bmp, and Shh signaling during the bud and cap stages of tooth development. Mice lacking *Lrp4* or *Sostdc1* have multiple dental anomalies including supernumerary incisors and molars. We clinically, radiographically, and molecularly investigated 94 Thai patients with mesiodens. *Lrp4* mutant mice were generated to study the effects of aberrant *Lrp4* expression in mice. Our study showed for the first time that heterozygous genetic variants in *LRP4* are contributing factors in seven patients with mesiodens, oral exostoses, and root maldevelopments. In addition, supernumerary incisors were observed in *Lrp4* mutant mice, supporting the phenotypes we found in our patients. The formation of mesiodens patients and supernumerary incisors in mice were possibly as a result of altered WNT/β-catenin-BMP-SHH signaling.

**Abstract:**

Background: Low density lipoprotein receptor-related protein 4 (LRP4; MIM 604270) modulates WNT/β-catenin signaling, through its binding of WNT ligands, and to co-receptors LRP5/6, and WNT inhibitors DKK1, SOSTDC1, and SOST. LRP4 binds to SOSTDC1 and WNT proteins establishing a negative feedback loop between Wnt/β-catenin, Bmp, and Shh signaling during the bud and cap stages of tooth development. Consistent with a critical role for this complex in developing teeth, mice lacking *Lrp4* or *Sostdc1* have multiple dental anomalies including supernumerary incisors and molars. However, there is limited evidence supporting variants in *LRP4* in human dental pathologies. Methods: We clinically, radiographically, and molecularly investigated 94 Thai patients with mesiodens. *Lrp4* mutant mice were generated in order to study the effects of aberrant *Lrp4* expression in mice. Results: Whole exome and Sanger sequencing identified three extremely rare variants (c.4154A>G, p.Asn1385Ser; c.3940G>A, p.Gly1314Ser; and c.448G>A, p.Asp150Asn) in *LRP4* in seven patients with mesiodens. Two patients had oral exostoses and two patients had root maldevelopments. Supernumerary incisors were observed in *Lrp4* mutant mice. Conclusions: Our study implicates heterozygous genetic variants in *LRP4* as contributing factors in the presentation of mesiodens, root maldevelopments, and oral exostoses, possibly as a result of altered WNT/β-catenin-BMP-SHH signaling.

## 1. Introduction

Tooth formation is a highly orchestrated process involving a well-characterized series of reciprocal interactions between the ectoderm-derived dental epithelium and the underlying neural crest-derived ectomesenchyme. The process of tooth formation begins during the seventh week of gestation in human or 11th day of embryogenesis in mouse [1] with the condensation of neural crest-derived mesenchyme subjacent to regions of the oral ectoderm. The interaction between the ectodermal placodes and the mesenchyme is critical for both tooth morphogenesis and differentiation of the various dental cell types that produce the specialized mineralized extracellular matrices: enamel, dentin, and cementum. Mesenchymal cells differentiate into the dentin-secreting odontoblasts and cementum-secreting cementoblasts, while enamel is secreted by the ameloblasts. The process of tooth formation is regulated by a number of evolutionarily conserved molecular signaling pathways, including WNT, SHH, BMP, and FGF [1].

The initiation of WNT/β-catenin signaling requires the binding of a WNT ligand to a FZD receptor and a co-receptor, LDL receptor-related protein 5 or 6 (LRP5 or LRP6), forming a WNT-FZD-LRP5/6 complex [2]. The activity of WNT/β-catenin signaling is also modulated by the binding of WNT ligands to inhibitors such as DKK1, DKK2, KREMEN1, KREMEN2, SOST, and SOSTDC1 [2]. In addition, the binding of WNT inhibitors to LRP5 and LRP6 co-receptors also affects WNT/β-catenin signaling. Mechanistically, the fine-tuning of WNT/β-catenin signaling depends on the numbers of the receptors, WNT ligands, and WNT inhibitors and how they interact with each other.

Low density lipoprotein receptor-related protein 4 (LRP4; MIM 604270) is a member of large evolutionarily conserved LDL receptor family of transmembrane proteins. The important roles of LRP receptors are to regulate the lipoproteins in the extracellular fluids and deliver them into cells. In addition, they also function as direct signal transducers or modulators for a number of cellular signaling pathways. Lrp4 is a member of the LDL receptor family and ENU-induced *Lrp4* knockout mice die at birth of severe congenital malformations. LRP4 modulates WNT/β-catenin signaling, through its binding to WNT ligands, co-receptors LRP5/6, and the WNT inhibitors DKK1, SOSTDC1, and SOST [3,4]. LRP4 also shares conserved amino acid sequence with the extracellular EGF repeats 1 and 2 of LRP5 and LRP6, which also bind to WNT ligands and the WNT inhibitor SOSTDC1 [3]. During tooth development, *Lrp4* is expressed in epithelial cells [2]. Consistent with the established role for Wnt signaling in regulating tooth development, *Lrp4*-knockout mice have been reported to exhibit supernumerary incisors, molars, and fused molars, similar to those of *Sostdc1* mutant mice [3,5,6].

Mechanistically, Sostdc1 binds to Bmp protein prior to its binding to Lrp4, usurping Bmp protein before it reaches its receptors. This results in downregulation of both Bmp and Wnt/β-catenin signaling [3], and subsequent downregulation of Shh signaling. Therefore, Lrp4 and Sostdc1 establish a negative feedback loop between Wnt/β-catenin, Bmp, and Shh signaling during the bud and cap stages of developing teeth [1].

Mesiodens is the supernumerary tooth located in the premaxilla region. The prevalence of mesiodens is about 2% [7]. Recently, variants in *LRP5*, *LRP6,* and *WLS* have been reported to be implicated in mesiodens in humans [8,9,10]. Here, we report the first heterozygous missense variants in *LRP4* in patients presenting with mesiodens with or without root maldevelopments and oral exostoses. A causal role for these variants is supported by the presentation of supernumerary incisors in *Lrp4* knockout mice.

## 2. Materials and Methods

### 2.1. Patient Recruitment

This patient-focused study was conducted in accordance with the Declaration of Helsinki and national guidelines. Informed consent was obtained from the parents in accordance with the regulations of the Human Experimentation Committee of the Faculty of Dentistry, Chiang Mai University (certificate of approval number 71/2020).

Oral and radiographic examinations (panoramic radiography or periapical radiography) were performed on the cohort of 94 patients with mesiodens. Among the 94 patients with mesiodens, 64 (68.1%) were males and 30 (31.9%) were females. Seventy-eight patients (82.9%) had single mesiodens, while 16 (17.1%) of them had double mesiodentes. For the orientation of the mesiodens, we were able to retrieve the information from our record only of 64 mesiodentes; 43 (67.1%) had normal orientation, 20 (31.3%) were inverted, and 1 (1.6%) had transverse orientation. Regarding the eruption status, we were able to retrieve the information of 60 mesiodentes; 32 (53.3%) erupted and 28 (46.7%) were unerupted.

### 2.2. Whole Exome Sequencing, Mutation Analysis, and Bioinformatic Analyses

Genomic DNA was isolated from saliva using Oragene-DNA (OG-500) Kit (DNA Genotek, Ottawa, ON, Canada). Using the targeted capture kit, SureSelect V6 ((PR7000-0152; Agilent Technologies, CA, USA, whole exome sequencing (WES) was performed on all 94 patients with mesiodens (Macrogen Inc, Seoul, Korea). The average depth of sequencing for this cohort was 80×. We adopted genomics analysis toolkit (GATK) germline mutation workflow version 3.8.1 to identify variants for each sample; the alignment of the raw sequencing FASTQ file was carried out using BWA-mem with the human genome reference sequence GRCh37. Standard variant filtering pipelines based on allele frequency, CADD scores (>15) and pathogenicity algorithms were applied to identify rare variants of interest. Variant effect predictor (VEP) and the database of nonsynonymous functional prediction (dbNSFP) were used to computationally assign effects to the resulting variants of each individual. The annotated variant calling format (VCF) files were stored in our in-house database that allows us to query pathogenic variants according to different modes of segregation when taking into account a pedigree of the probands. Furthermore, variant allele frequencies were determined by comparing against public databases, including gnomAD, 1000G, GenomeAsia, and the recent Thai Reference Exome (T-Rex) database.

Sanger sequencing was performed to confirm the variants. The sequence primers used were as followed: Exon 7, forward: 5′-GCTCCACAAGCCTTCTCCTTA-3′; reverse: 5′-CCCTCTTGGGAAGAGATGGAG-3′. Exon 28, forward: 5′- TGGTCAGAACACAACCTCACC-3′; reverse: 5′-GCCAGCCACAAACAACTGGG-3′. Exon 35, forward: 5′-AGTGCCTTGCACGGATTTCT-3′; reverse: 5′-TGTCACTGTAAGTGGTGAGAGC-3′.

### 2.3. LRP4 Structural Analysis

The protein linear map was created based on annotation in the National Center for Biotechnology Information (NCBI) entry accession number NP_002325.2 and compared with the predicted structure. The mutation locations were placed in this map to identify which domains they may disrupt. The predicted human LRP4 structure was obtained from the AlphaFold2 database of human protein structures provided by the European Bioinformatics Institute (https://alphafold.ebi.ac.uk, accessed on 8 January 2023) accessed on January 8 2023 [11] (UniProt: O75096). The mutations were introduced in PyMol (Schrödinger LLC) and the lowest energy rotomer for the new amino acid selected for comparison. The interactions of the wild type and variant amino acid with surrounding amino acids were assessed by visualization and distance measurements in PyMol.

### 2.4. Lrp4 Knockout Mice

*Lrp4* mutant mice were generated as described by [12]. Briefly, disruption of *Lrp4* by homologous recombination was achieved by introducing a stop codon just upstream of the transmembrane segment. This prevents the production of a membrane-anchored receptor and abolishes any possibility of any residual functional activity through alternative splicing of the extracellular domain or the use of alternative promoters for transcription initiation. Mice were genotyped using PCR of genomic DNA extracted from tail as follows: MEJ155 (50-CCCAGCTGGGCCTCTGTGCACATTCCAATG-30) and MEJ166 (50-CCATGGCCTCTGCATTAGTTCTTGCTCTC-30) were used to selectively amplify the wild-type allele and MEJ156 (50-CTCTGAAAGGGATGCCCAGCTGGGCCTCTG-30) and MEJ267 (50 CGATGGCATAGCTGACTTA-30) were used to amplify the knockout allele. The mouse specimens were fixed using Bouin solution.

## 3. Results

### 3.1. Whole Exome Sequence Sequencing and Bioinformatic Analysis

**Figure 1:**
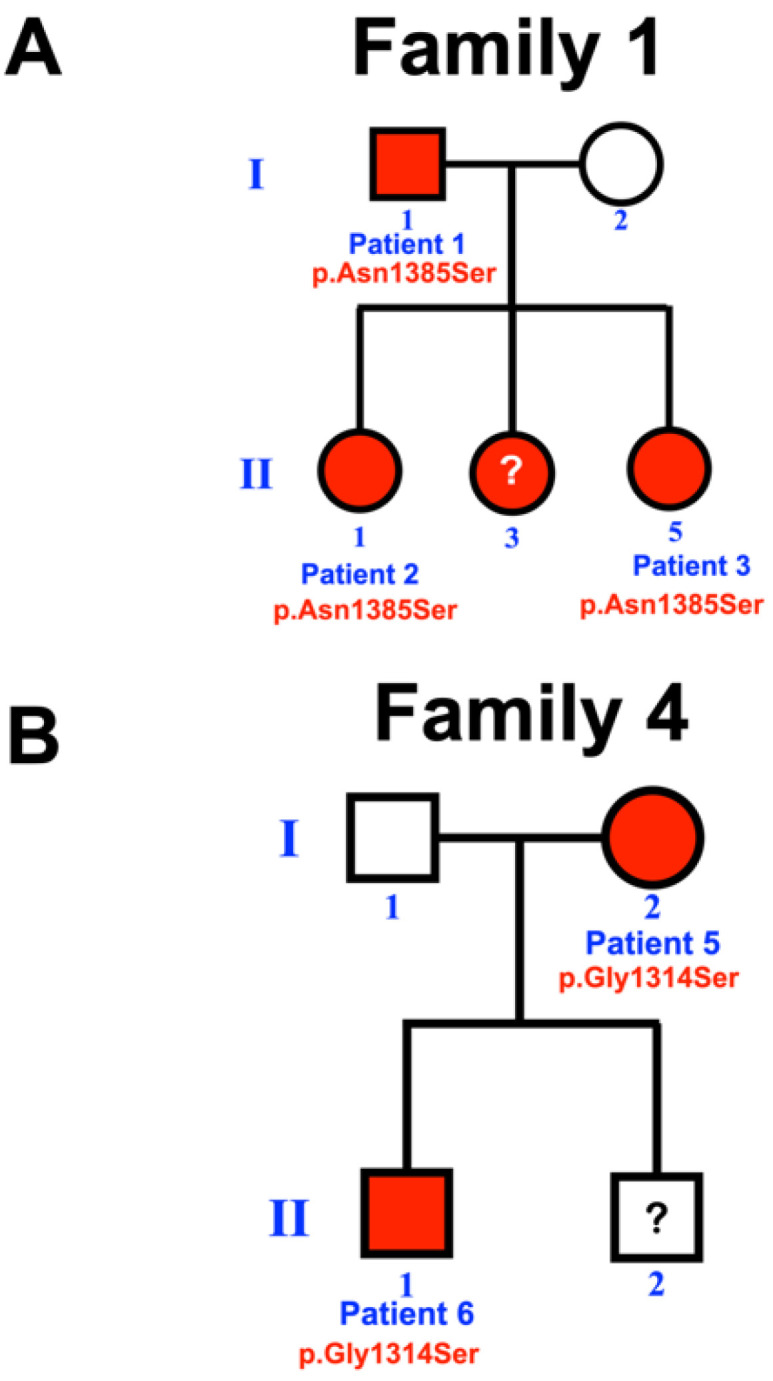
Pedigrees of (**A**) family 1 and (**B**) family 4. Patient II-3 of family1 had mesiodens but not available for genetic study. Patient II-2 of family 4 is not available for study.

**Figure 2:**
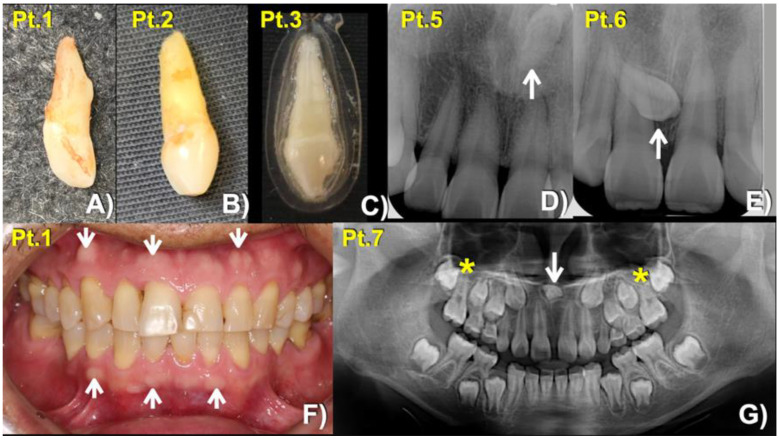
Mesiodens phenotypes. Extracted mesiodens of (**A**) patient 1, (**B**) patient 2. (**C**) patient 3. (**D**,**E**) Periapical radiographs show (**D**) Patient 5—Inverted mesiodens (arrow). (**E**) Patient 6—Unerupted mesiodens (arrow). (**F**) Patient 1—Buccal exostoses (arrows). (**G**) Panoramic radiograph showing inverted mesiodens (arrow) and unseparated roots of the maxillary first permanent molars (asterisks).

**Figure 3:**
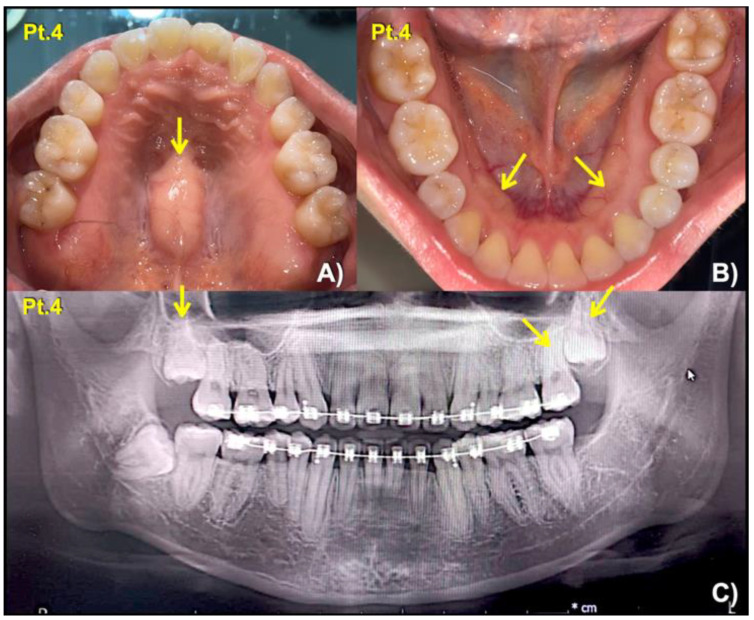
Patient 4. (**A**) Torus palatinus (arrow). (**B**) Torus mandibularis (arrows). (**C**) Panoramic radiograph showing long roots of mandibular permanent canines, short roots of second premolars, and unseparated roots of second and third permanent molars (arrows).

**Figure 4:**
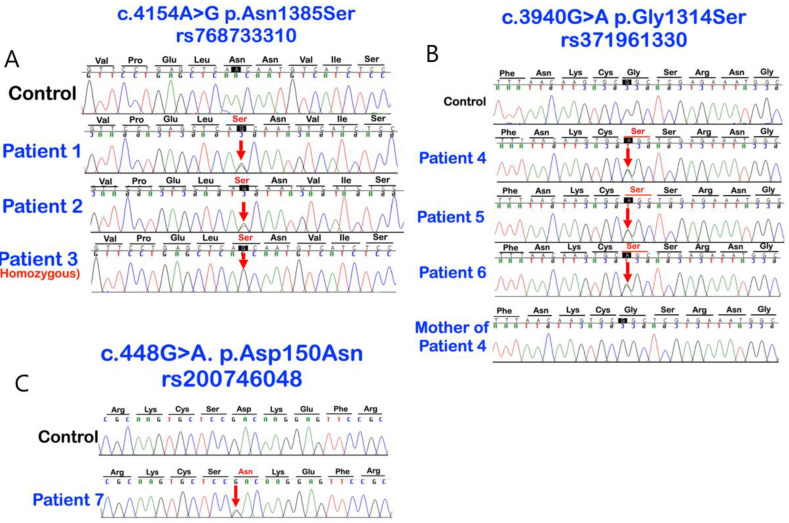
Sequence chromatograms of *LRP4* variants. (**A**) The c.4154A > G, p.Asn1385Ser (rs768733310) variant in patients 1–3. Patient 3 is homozygous for the variant; (**B**) The c.3940G>A; p.Gly1314Ser (rs371961330) variant in patients 4–6; and (**C**) the c.448G>A; p.Asp150Asn (rs200746048) variant in patient 7.

Our bioinformatic pipeline identified extremely rare missense variants in *LRP4* in three of the 94 patients: chr11:g.46896426T > C, c.4154A > G, p.Asn1385Ser (rs768733310); chr11:g.46896640C > T, c.3940G > A, p.Gly1314Ser (rs371961330); and chr11:g.46921037C > T, c.448G > A, p.Asp150Asn (rs200746048) with CADD > 15 (Table 1). Extended testing of available family members ultimately identified each variant in other affected family members. In total, seven individuals (7.4%) affected with mesiodens from four unrelated families were found to carry the respective rare variants. Patient 3 was homozygous for the p.Asn1385Ser variant. The other six patients were heterozygous for the variants (Figure 1, Figure 2, Figure 3 and Figure 4; Table 1). Assessment of the gnomAD databases revealed allele frequencies of 0.00001768 (c.4154A>G, p.Asn1385Ser), 0.00009579 (c.3940G>A, p.Gly1314Ser), and 0.00002787 (c.448G>A, p.Asp150Asn). None of the variants were found in our in-house exome database of 925 individuals of Thai ancestry. All three amino acid variants are predicted to be disease-causing and pathogenic by MutationTaster (https://www.mutationtaster.org) accessed on 8 January 2023 and Deleterious Annotation of genetic variants using Networks [13], respectively.

### 3.2. Protein Models

**Figure 5:**
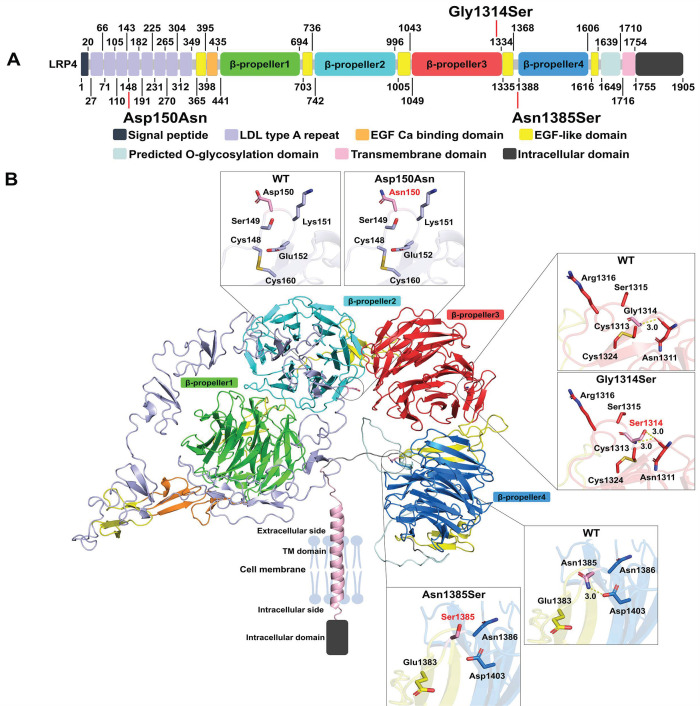
Structural context of LRP4 mutations. (**A**) Map of structural domains and mutations within the LRP4 protein sequence. Structural domains are coded by color as indicated. The residue numbering is based on accession number NCBI: NP_002325.2. The starting position of each structural domain is marked on the bottom and the ending position on the top. (**B**) Three-dimensional model of the LRP4 structure predicted by AlphaFold2 with structural domains in the same colors as in part A. Magnified views of the wild type and mutated amino acids and surrounding amino acids in stick representation are shown to the side to indicate the molecular environment. Possible polar interactions are shown as dashed lines. Oxygen atoms are shown in red, nitrogen in blue and sulfur atoms in yellow, while carbon atoms are colored according to the domain color. The intracellular domain has no predicted structure and is shown schematically as a box.

The LRP4 protein is composed of a signal peptide, eight low-density lipoprotein receptor class A domains (LDLa), five epidermal growth factor (EGF)-like domains, four β-propeller domains, a predicted O-glycosylation site, a transmembrane domain, and an intracellular domain [14] (Figure 5). The p.Asp150Asn variant is situated in the fourth LDLa repeat and close to key binding surface residues (Figure 5A). Protein modeling suggests this variant would decrease the surface negative charge, which may disrupt the binding with SOSTDC1 (Figure 5B). p.Gly1314Ser is found within the third β-propeller of the extracellular region of the receptor (Figure 5A). Generally, Gly residues are highly conserved, since they fit in tight spaces in protein structures and provide flexibility. Increasing the residue size at position 1314 with a Ser, which can hydrogen bond to the nearby Asn1311 residue, may change the shape and flexibility of this loop in β-propeller 3 and affect its binding to ligands such as SOST and SOSTDC1 [15] (Figure 5B). The p.Asn1385Ser variant resides in the YWTD region between EGF-like domain 4 and the LDL class B repeat 16 of the larger β-propeller domain 4 (Figure 5A). The change from Asn to Ser at 1385 leads to a smaller side chain with fewer potential interactions, including a predicted hydrogen bond between Asn1385 and Asp1403, which may affect the stability of this region and its interactions with protein ligands (Figure 5B).

### 3.3. Lrp4 Knockout Mice and Supernumerary Incisors

**Figure 6:**
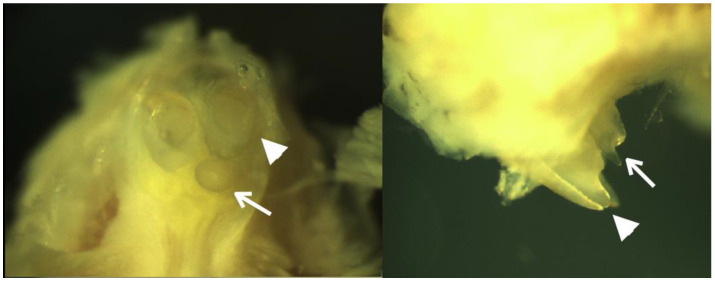
*Lrp4* mutant mice. Arrowheads indicating endogenous incisors, while arrows indicating supernumerary incisors.

Examination of *Lrp4* mutant mice found supernumerary incisors (mesiodens) which were located on the lingual sides of the endogenous maxillary incisors (Figure 6), as previously reported.

## 4. Discussion

To date, pathogenic variants in *LRP4* have been described in multiple conditions. Biallelic variants in *LRP4* are implicated in autosomal recessive Cenani-Lenz syndactyly syndrome (CLS; MIM 212780) and autosomal recessive congenital myasthenic syndrome (CMS17; MIM 616304). Monoallelic and biallelic variants in *LRP4* are also associated with sclerosteosis 2 (SOST2; MIM 614305), with patients with biallelic variants presenting with a more severe and complex phenotype [16,17]. Here, we describe three distinct rare missense variants in *LRP4* variants in seven patients from four unrelated Thai families. All of our patients presented with very mild or often unnoted phenotypes, including mesiodens, oral exostoses, and root maldevelopment, although one patient (patient 4) also had torus palatinus and torus mandibularis. Six of the patients were heterozygous for the variants, while one patient—patient 3—was found to be homozygous for the p.Asn1385Ser variant. No other rare variants in known tooth-associated genes (including *WNT10A, WNT10B, PAX9, AXIN2, MSX1, LRP5, LRP6, WLS, BMP4, GREM2, TFAP2B, TSPEAR, EDA, EDAR, EDARADD, PITX2, EVC, EVC2, COL1A2, ANTXR1, FGF10, SMOC2, KREMEN1, KDF1, ATF1, DUSP10,* and *CASC8* [8,9,10,18]) were identified in any of these patients.

We do not believe these are co-incidental findings. Rather, we hypothesize that these three variants are “predisposing factors” to mesiodens, oral exostoses, and root maldevelopment for the following reasons. Firstly, the p.Asn1385Ser, p.Gly1314Ser, and p.Asp150Asn variants are extremely rare with allele frequencies of 0.00001768, 0.00009579, and 0.00002787, respectively. Secondly, neither variant was found in our in-house exome database of 925 individuals of Thai ancestry, excluding each as a common ethnic variant. Thirdly, these variants are predicted to be disease-causing and pathogenic by MutationTaster and Deleterious Annotation of genetic variants using Neural Networks, respectively. Fourthly, three patients with mesiodens from two unrelated families (patients 4-6; families 2 and 3) carried the same rare variant (p.Gly1314Ser), supportive of its pathogenicity. Fifthly, a causal role for these variants is supported by the presentation of supernumerary incisors in *Lrp4* knockout mice in our study and the previously reported one [3]. Lastly, tooth agenesis has been reported in a patient with a compound heterozygous mutation in *LRP4*, directly implicating this gene in dental anomalies in humans [14].

In a 100 species multi-alignment, each of the altered amino acid residues Asp150, Gly1314, and Asn1385 was found to be highly conserved. Asn1385 was almost invariant across all 100 species, while Asp150 showed the least conservation (with variants found in birds, marsupials, and a few fishes—although none with the same Asn substitution) (https://genome.ucsc.edu) accessed on 8 January 2023. For Gly1314, eight of the 10 species with differing amino acids in this position had the same Serine substitution as seen in our patients (Figure 4). These species included whales, dolphins, turtles and a few fish, all of which have very different shapes and numbers of teeth to humans. It is noteworthy that most dolphins have one set of teeth that are conical, similar to mesiodens [19] (Figure S1).

**Figure 7:**
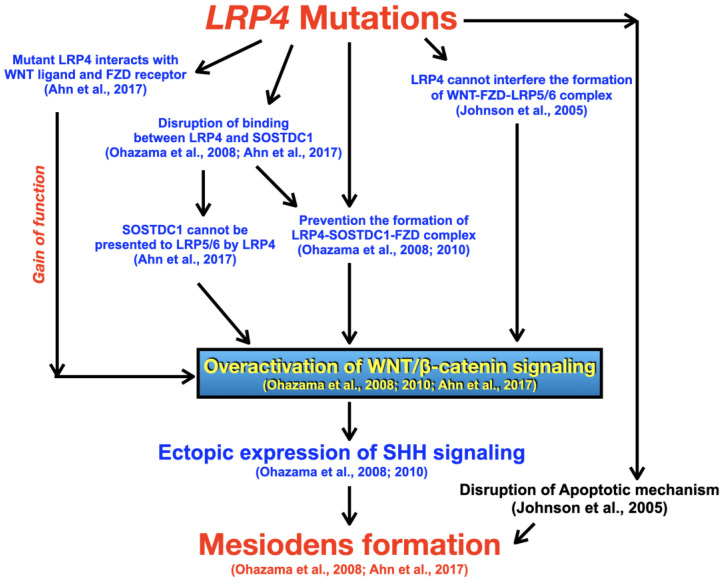
Hypothetical flowchart of pathogenetic pathways for mesiodens formation as a result of *LRP4* variants. When the mutant Lrp4 does not properly interact with Sostdc1 as a result of mutation, it upregulates Wnt/β-catenin signaling by forming the Wnt-Fzd-Lrp4 complex. The resulting increase in WNT/β-catenin signaling and/or broadening of the placodal regions is responsible for the subsequent formation of the supernumerary teeth. Overactivation of WNT/β-catenin signaling leading to ectopic expression of SHH signaling is the key to mesiodens formation [3,4,6,12].

Downregulation of WNT/β-catenin signaling has long been implicated in agenesis of teeth or microdontia and overactivation of WNT/β-catenin signaling results in supernumerary tooth formation [1]. Lrp4 may function as a Wnt/β-catenin activator or inhibitor, depending on its interactions with Sostdc1 or Wnt ligands [4]. Of note, *Sostdc1* has been shown to have important role in determining skin appendage placode number [20]. In order to develop the proper number of teeth, Lrp4 needs to bind to Sostdc1 in order to downregulate Wnt/β-catenin and Bmp signaling to define the size and position of the dental placodes, and thus prevent the development of supernumerary teeth [3,4]. Sostdc1 might also be presented to Lrp5/6 via Lrp4, resulting in displacement of Wnt ligands and inhibition of Wnt/β-catenin signaling [4]. In addition to being a Wnt inhibitor, *Sostcd1* is also a Shh target gene, supporting a negative feedback loop between Wnt and Shh signaling, which specifies the tooth-forming fields in embryonic oral ectoderm [1,4]. Sostdc1 also antagonizes Bmp activity in oral epithelial cells and this requires the presence of Lrp4 [3]. When Lrp4 does not interact with Sostdc1, it upregulates Bmp and Shh activity and well as directly activating Wnt/β-catenin signaling by forming the Wnt-Fzd-Lrp4 complex [4]. The resulting increase in WNT/β-catenin signaling and/or broadening of the placodal regions is responsible for the subsequent supernumerary teeth in the diastemal areas [4] (Figure 6 and Figure 7). The supernumerary incisors in *Lrp4* knockout mice (Figure 6) implicates it in the regulation of tooth number [3] and suggest that mesiodens might manifest slightly differently in mice. We therefore hypothesize that the *LRP4* variants detected in our patients with mesiodens would represent partial loss of function alleles that predispose individuals to mesiodens (Figure 7).

It is worth noting that patients 1 and 4 also presented with oral exostoses while other root maldevelopments were seen in patients 4 and 7. These are also likely the effects of disruptive WNT/β-catenin signaling, given the importance of this pathway on root development. This hypothesis is supported by the findings of these phenotypes in the patients with genetic variants in the *WLS* [10] and WNT coreceptor *LRP5* [8,21] or *LRP6* [9,22].

Curiously, one of our patients, patient 3, was found to be homozygous for one variant (p.Asn1385Ser) and did not appear to be more severe in their presentation than heterozygous family members. This may simply be indicative of a partial loss of function variant that can be modified by variants in other gene pathways. In this regard, individuals carrying the p.Gly1314Ser variant showed marked differences in severity. Thus, we feel these LRP4 variants are likely primary ‘predisposing’ risk alleles.

## 5. Study Limitations

Our cohort consists of 94 patients with mesiodens phenotype. However, we only have DNA samples and dental information of the affected patients who came for oral and radiographic examinations. We are aware that it would have been ideal if we had each patient’s unaffected family members to study co-segregation between genotype and phenotype. It would also have strengthened the association of heterozygous variants in *LRP4* and the mesiodens phenotype.

## 6. Conclusions

Our study shows for the first time implicating rare genetic variants in *LRP4* as risk alleles for mesiodens, root maldevelopments, and oral exostoses. This association is supported by the findings of supernumerary incisors in *Lrp4* mutant mice. We surmise that the resultant reduced LRP4 function disrupts BMP and WNT/β-catenin signaling in the oral ectoderm and may result in splitting of the *SHH*-expressed signaling center, forming two epithelial invaginations, and subsequent mesiodens formation. This conclusion is also consistent with our recent report of heterozygous variants in genes in WNT/β-catenin signaling, including *WLS*, *LRP5,* and *LRP6* in other patients with mesiodens, tooth agenesis, root malformation, and oral exostoses [8,9,10].

## Figures and Tables

**Table 1:** Patients with *LRP4* Variants and their phenotypes. All patients have mesiodens with or without root maldevelopments and oral exostoses. Where known, the orientation and eruption status are shown for each. Patient 3 was homozygous for the p.Asn1385Ser variant, the other six patients were heterozygous for *LRP4* variants. Patients 2 and 3 are daughters of patient 1. Patient 6 is the son of patient 5. Genomic coordinates are based on GRCh37. RefSeq accession numbers NM_002334.4; NP_002325.2 Genomic coordinates are given relative to GRCh37v1.6.

Patients Gender/Age	Families	Phenotypes	*LRP4* Variants	DANN Score	CADD Scores (GRCh38)
1 (Male: 52 Yr)	1	Mesiodens (Conical; erupted) buccal exostoses	c.4154A > G; p.Asn1385Ser chr11:g.46896426T > C rs768733310, MAF: 0.00001768	0.9926 (pathogenic)	22.3
2 (Female: 33 Yr)		Mesiodens (Conical; erupted)			
3 (Female: 17 Yr) Homozygous		Mesiodens (Conical; erupted)			
4 (Female: 30 Yr)	2	Mesiodens (Conical; unerupted) long roots of mandibular canines, short roots of second premolars, unseparated roots of second and third molars, torus mandibularis, and torus palatinus	c.3940G > A; p.Gly1314Ser chr11: g.46896640 C > T rs371961330 MAF = 0.00009579	0.9966 (pathogenic)	18.11
5 (Female: 41 Yr)	3	Mesiodens (Inverted; unerupted)			
6 (Male: 13 Yr)		Mesiodens (Unerupted, tuberculate)			
7 (Female: 9 Yr)	4	Mesiodens (inverted; unerupted) unseparated roots of the maxillary first molars	c.448G > A; p.Asp150Asn chr11:g.46921037C > T rs200746048, MAF: 0.00002787	0.9925 (pathogenic)	22.3

## Data Availability

Not applicable.

## Supplementary Materials

The following supporting information can be downloaded at: https://www.mdpi.com/article/10.3390/biology12020220/s1, Figure S1: Amino acid Conservation of amino acid residues. (A) Asn1385. (B) Gly1314. (C) Asp150. The amino acid residues Asp150, Gly1314, and Asn1385 are conserved from humans to monkeys. The amino acid residue Gly1314, our patients with mesiodens had amino acid Ser at this position as dolphins and whales do.
