# Supplementary material for: Rare Variants in LRP4 Are Associated with Mesiodens, Root Maldevelopment, and Oral Exostoses in Humans
Source: Biology (Basel). 2023 Jan 30;12(2):220. doi: 10.3390/biology12020220 (PMC9952772; doi:10.3390/biology12020220)
Supplement: Supplementary file 1 [file biology-12-00220-s001.zip › biology-2099596-supplementary.pdf]

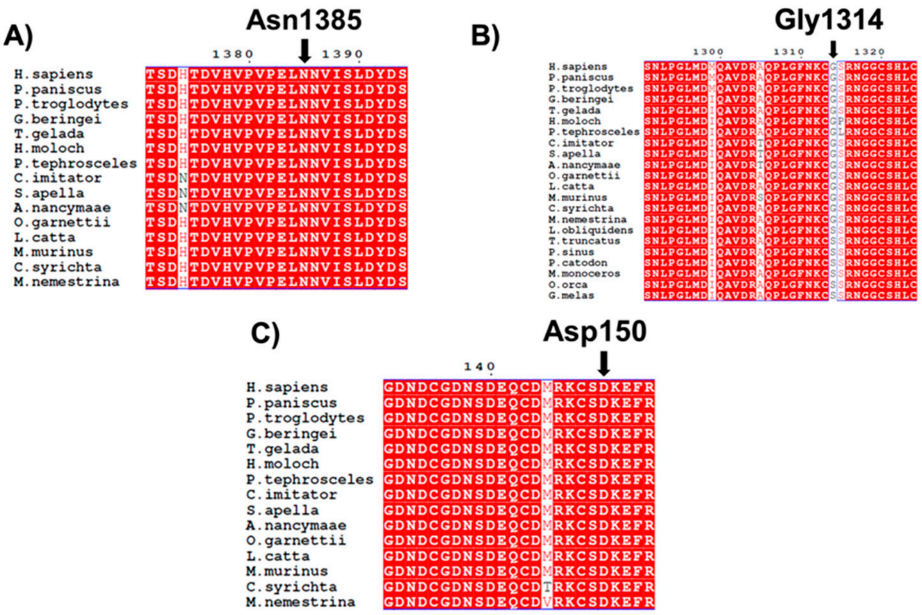

Figure S1. Amino acid Conservation of amino acid residues. (A) Asn1385. (B) Gly1314. (C) Asp150. The amino acid residues Asp150, Gly1314, and Asn1385 are conserved from humans to monkeys. The amino acid residue Gly1314, our patients with mesiodens had amino acid Ser at this position as dolphins and whales do.
